# Nephrotoxicity of Evodiamine in Mice: Mechanistic Insights from Integrated Network Toxicology and Transcriptomic Profiling

**DOI:** 10.3390/ijms27093793

**Published:** 2026-04-24

**Authors:** Xuehua Zhang, Yue Pan, Yuanyuan Xiao, Ziyan Wu, Huilan Yang, Yanjun Liu, Yan Wang, Tianqi Chen, Wenchao Tang

**Affiliations:** 1 School of Basic Medicine, Guizhou University of Traditional Chinese Medicine, Guiyang 550025, China; 18386997042@163.com (X.Z.); pany2026@163.com (Y.P.); yuanyuanxiao1991@163.com (Y.X.); wuziyan2026@163.com (Z.W.); yanghuilan2026@163.com (H.Y.); tq9314026@163.com (Y.L.); wangyanpiano@163.com (Y.W.); chentianqi@163.com (T.C.); 2 School of Traditional Chinese Medicine Health Preservation, Guizhou University of Traditional Chinese Medicine, Guiyang 550025, China

**Keywords:** network toxicology, transcriptomics, evodiamine, nephrotoxicity

## Abstract

The aim of this study was to evaluate the nephrotoxicity and molecular mechanism of Evodiamine (EVO). We combined RNA sequencing (RNA-seq) and network toxicology (NT) screening of potential target genes and signaling pathways, used molecular docking to validate core targets, and detected the mRNA expression of the key genes through quantitative real-time polymerase chain reaction (qRT-PCR). After exposure to EVO, body weight of mice decreased significantly, and the levels of renal index, Blood Urea Nitrogen (BUN) and Creatinine (Cr) were significantly increased, with varying degrees of pathological damage to the kidneys. NT identified 125 intersecting targets of EVO exposure related to kidney injury, including AKT1, TNF, TP53, etc. Among the 2888 differentially expressed genes obtained from RNA-seq, 504 genes were up-regulated and 2384 genes were down-regulated. By integrating NT and RNA-seq, 24 intersecting targets were identified. Among them, TRPV1, NOS3, HSP90AA1, and PPARG were selected for molecular docking validation. The results indicated that EVO had the highest affinity for PPARG (−8.07 kcal/mol). The qRT-PCR results indicated that the expression of the *Pparg* and *Hsp90aa1* genes was significantly down-regulated, and the expression of the *Nos3* and *Trpv1* genes was significantly up-regulated. Immunohistochemistry further confirmed that EVO inhibited the expression of HSP90AA1 and PPARG, while enhancing that of TRPV1 and NOS3. Kyoto Encyclopedia of Genes and Genomes (KEGG) pathway enrichment analysis suggested that EVO-induced nephrotoxicity is related to signaling pathways such as inflammatory mediator regulation of TRP channels, the PPAR signaling pathway, and the Apelin signaling pathway. In summary, the nephrotoxic effect of EVO may be related to the inhibition of the PPARG signaling pathway, the activation of the TRPV1 channel, the reduction in HSP90AA1 expression, and the imbalance of the Apelin-NOS3 pathway. This study provides a theoretical reference for clarifying the potential mechanism of renal injury caused by EVO and guiding its safe use.

## 1. Introduction

*Evodia rutaecarpa* is a dry nearly mature fruit from Rutaceae plants. It is traditionally used to alleviate pain, dispel cold, arrest vomiting, soothe the liver and dry dampness [1]. Modern pharmacological research has demonstrated that *Evodia rutaecarpa* exerts anti-tumor, antibacterial, anti-inflammatory and antioxidant effects [2], and is clinically used to treat abdominal pain, bloating, vomiting and diarrhea [3]. Evodiamine (EVO) is the main alkaloid component of *Evodia rutaecarpa* [4]. EVO exhibits antioxidant, anti-tumor and anti-inflammatory activities, and is widely used to manage headache, abdominal pain, amenorrhea, dysmenorrhea and postpartum hemorrhage [5,6].

However, since the *Shen Nong’s Herbal Classic*, *Evodia rutaecarpa* has been recorded to be a slightly toxic Chinese herb, and is listed in the *Chinese Pharmacopeia* as a mildly toxic medicinal herb [7]. *Evodia rutaecarpa* has a relatively low toxicity. Although its general acute toxicity is low, excessive dosage may induce headache, abdominal pain, visual disturbance and abortion [8]. EVO serves as both the active constituent and the toxic component of *Evodia rutaecarpa* [9]. Its clinical use carries potential risks of liver and kidney injury [10]. Studies have shown that high-dose EVO causes cardiorenal damage and reduces organ function in zebrafish [10,11]. In cell experiments, different concentrations of EVO significantly reduce HK-2 cell viability, resulting in reduced cell size and number [10]. In animal experiments, EVO can cause mild edema of renal tubular epithelial cells, nuclear pyknosis, hyperchromasia, and even degeneration and necrosis [10]. However, the precise molecular mechanism underlying EVO-induced nephrotoxicity remains largely unclear, which greatly restricts its safe clinical application.

Network toxicology is an emerging field rooted in the foundational concepts of network pharmacology. This discipline characterizes the toxicological properties of pharmaceutical agents, constructs targeted network frameworks, and uses network-based analytical approaches to predict the toxic potential of drugs and their components, ultimately revealing the adverse toxic reactions they cause [12]. Its application in studying the molecular mechanisms of toxicity is characterized by speed, convenience, integrity and comprehensiveness [13]. Molecular docking is a well-established computer-aided structure-based method that predicts and validates ligand–target binding interactions and affinities at the molecular level [14]. When applied to toxicology, it helps reveal toxin–biomolecule interactions and clarify toxicity mechanisms [15]. Liu et al. [16] identified key targets and biological pathways involved in cantharidin-induced testicular toxicity using network toxicology and molecular docking. Transcriptomics offers a comprehensive overview of gene expression profiles, enabling researchers to characterize shifts in gene activity across diverse cellular, tissue, or organismal contexts and uncover critical insights into gene regulatory circuits and disease mechanisms [15]. As high-throughput biotechnologies have advanced, integrating network toxicology with omics-based strategies has become a common approach for identifying the toxicological targets of chemical agents [17]. Yang et al. [17] integrated network toxicology with transcriptomic profiling to explore the molecular targets driving the hepatotoxicity of Benzo[a]pyrene exposure. Notably, EVO-induced nephrotoxicity involves complex biological processes and multi-target regulation. Network toxicology enables systematic prediction of potential toxic targets and pathways but relies on database predictions and lacks experimental support from actual gene expression changes. In contrast, transcriptomics genuinely reflects genome-wide transcriptional alterations in kidney tissue induced by EVO. Therefore, the integrated strategy of network toxicology and transcriptomics is particularly suitable for investigating EVO-induced nephrotoxicity.

Accordingly, we hypothesize that EVO exerts its nephrotoxic effects by regulating specific key targets and signaling pathways related to renal injury. To test this hypothesis, we established an in vivo mouse model of EVO-induced kidney damage, combined network toxicology and transcriptomics to identify potential core targets and pathways, and verified them using molecular docking, qRT-PCR, and immunohistochemistry. This study provides a theoretical basis for further exploration of the molecular mechanism underlying EVO-induced renal toxicity.

## 2. Results

### 2.1. Toxicity Evaluation of Evodiamine (EVO) In Vivo

**Figure 1:**
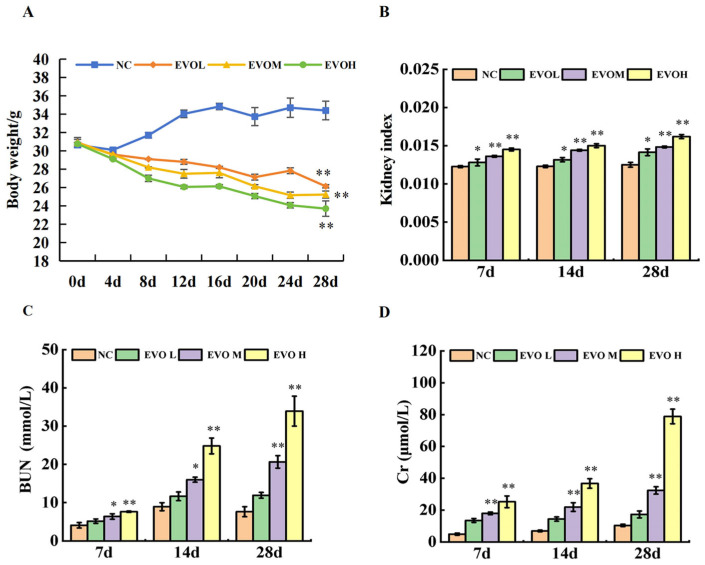
Evodiamine (EVO) induces nephrotoxicity in vivo. (**A**) Twenty-eight-day weight changes in mice; (**B**) renal index; changes in serum renal function indicators BUN (**C**) and Cr (**D**). Note: NC: normal control group; EVOL: EVO low-dose group (10 mg/kg); EVOM: EVO medium-dose group (20 mg/kg); EVOH: EVO high-dose group (40 mg/kg). N = 6, * represents significance compared to the NC group, * indicated *p* < 0.05, ** indicated *p* < 0.01.

Body weight changes in mice over 28 days of treatment are shown in Figure 1A. Compared with the normal control (NC) group, weight gain was significantly inhibited in the EVO groups over the 28 days (*p* < 0.01). Especially on the 20th day of administration, the EVO high-dose (EVOH) group exhibited a substantial decrease in body weight. The renal index changes in mice are illustrated in Figure 1B. By the 28th day of treatment, a statistically significant elevation in renal index was observed (*p* < 0.05, *p* < 0.01). Blood Urea Nitrogen (BUN) and Creatinine (Cr) levels are shown in Figure 1C,D. Compared with the NC group, the EVO treatment groups exhibited statistically significant increases in both BUN and Cr levels (*p* < 0.05, *p* < 0.01). These results collectively indicate that EVO exerts significant nephrotoxicity in vivo.

### 2.2. Histopathological Examination of Mouse Renal Tissues

**Figure 2:**
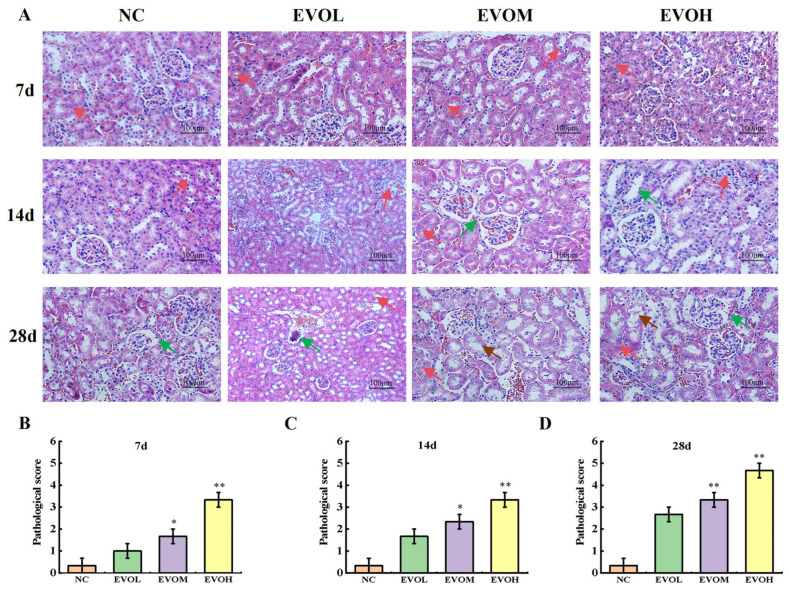
Pathological section of renal tissue. (**A**) Pathological changes in mice’s kidney (Hematoxylin and Eosin staining, ×200); (**B**–**D**) indicates renal pathology scores at 7d, 14d, and 28d, respectively (the red arrows represent inflammatory cell infiltration; the brown arrow indicates necrosis of the renal tubules; the green arrow indicates tubular cystic dilation of the renal tubules). Note: NC: normal control group; EVOL: EVO low-dose group (10 mg/kg); EVOM: EVO medi-um-dose group (20 mg/kg); EVOH: EVO high-dose group (40 mg/kg). N = 6, * represents significance compared to the NC group, * indicated *p* < 0.05, ** indicated *p* < 0.01.

Histological changes in the kidney tissues of mice from different treatment groups are depicted in Figure 2A. In the NC group, renal tissue displayed normal structure, with no inflammatory cell infiltration, patent capillary lumina, and no dilatation of the glomerular capsule. On treatment day 7, the EVO groups showed disorganized renal histology, irregular glomerular morphology and occasional inflammatory cell infiltration. On treatment day 14, glomerular structure in EVO groups was disorganized, with mild tubular dilation, increased inflammatory infiltration, and focal tubular necrosis. On treatment day 28, EVO groups showed marked glomerular capsule dilatation, extensive inflammatory cell infiltration, tubular swelling, and severe cellular edema, degeneration, and necrosis. The pathological injury scores are shown in Figure 2B,D. The NC group maintained an extremely low score of 0.33 ± 0.33 at 7, 14, and 28 days, indicating no significant renal injury; in contrast, the EVO dose groups exhibited a significant time- and dose-dependent increase in scores. On treatment day 7, the EVO low-dose group (EVOL), EVO medi-um-dose (EVOM), and EVOH groups scored 1.00 ± 0.58, 1.67 ± 0.33, and 3.33 ± 0.33, respectively, with renal injury observed in the EVO-treated groups. On treatment day 14, the scores rose to 1.67 ± 0.33, 2.33 ± 0.33, and 3.33 ± 0.67 for EVOL, EVOM, and EVOH groups, with further worsening of injury in the medium and high-dose groups. On treatment day 28, scores reached 2.67 ± 0.33, 3.33 ± 0.67, and 4.67 ± 0.33 in the EVOL, EVOM, and EVOH groups. Compared with the NC group, the pathological injury scores of all EVO groups were significantly increased on days 7, 14, and 28 (*p* < 0.05, *p* < 0.01). On day 28, the EVOH group exhibited severe toxic damage, with its score being more than 13 times higher than that of the NC group.

### 2.3. Targets of EVO-Induced Renal Toxicity

**Figure 3:**
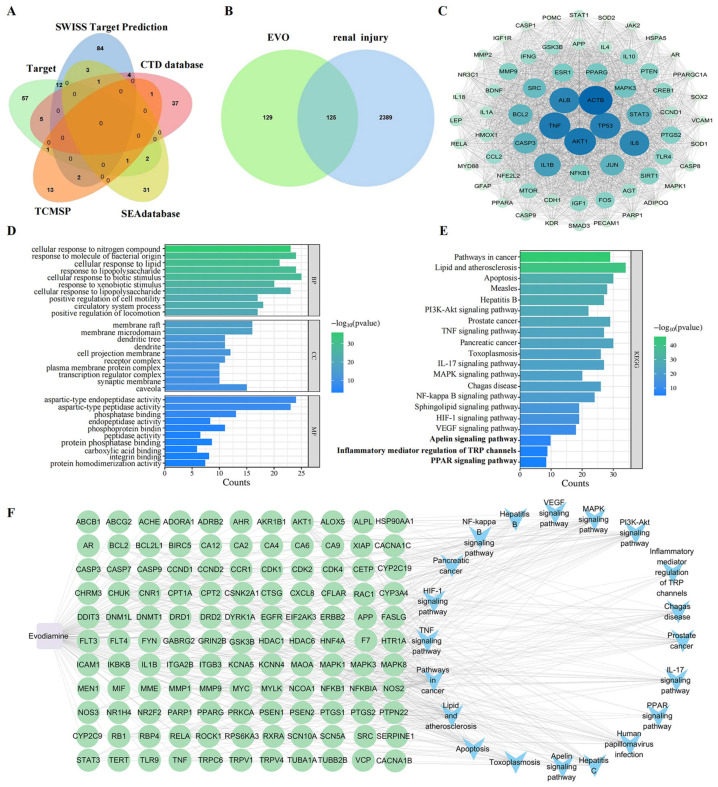
Network toxicology analysis of the mechanism of nephrotoxicity induced by EVO. (**A**) EVO targets collected from five databases; (**B**) target intersection Venn diagram of EVO and renal injury; (**C**) PPI network map of the target of EVO-induced nephrotoxicity; (**D**) GO functional enrichment analysis of 125 intersecting targets; (**E**) KEGG pathway analysis of 125 intersecting targets; (**F**) EVO-target-pathway diagram.

A total of 254 targets for EVO were identified by collecting data from multiple databases, followed by screening and duplicate removal (Figure 3A). In addition, 2514 targets associated with renal injury were retrieved from the GeneCards database. EVO targets and renal injury targets were uploaded to Venny 2.0 software, yielding 125 overlapping targets (Figure 3B).

#### 2.3.1. Protein–Protein Interactions (PPI) Network Analysis

To investigate PPI among overlapping targets, we analyzed data using the STRING database and visualized the PPI network with Cytoscape 3.10.1. The Degree value was employed to define both the size and color of each node in the network. Node size and color intensity were directly proportional to the Degree value, with higher values resulting in larger nodes, which signified a more important functional role of the corresponding target. The targets with the top ten highest Degree scores were identified as AKT1, TNF, TP53, ALB, ACTB, IL6, IL1B, STAT3, CASP3 and JUN (Figure 3C). AKT1, as a core node in the PI3K/AKT pathway, primarily regulates the survival, apoptosis, and oxidative stress response of renal tubular epithelial cells, with its abnormal activation or inhibition directly involved in the progression of nephrotoxic injury. TNF, IL6, IL1B, and STAT3 are classic pro-inflammatory regulatory molecules that drive renal inflammatory infiltration, tubular injury, and interstitial fibrosis, making them the most representative inflammatory targets in nephrotoxic mechanisms. TP53 and CASP3 jointly mediate the apoptosis signaling pathway, playing a critical role in drug-induced necrosis and apoptosis of renal tubular epithelial cells. ALB is primarily involved in maintaining the integrity of the glomerular filtration barrier, and its dysfunction is closely associated with proteinuria and glomerular structural damage. ACTB and JUN, on the other hand, participate in cytoskeletal remodeling and stress transcriptional regulation, respectively, and are highly correlated with renal tissue structural damage and functional disorders.

#### 2.3.2. Gene Ontology (GO) Functional Enrichment Analysis and Kyoto Encyclopedia of Genes and Genomes (KEGG) Enrichment Analysis

The 125 overlapping targets were analyzed using Metascape for GO and KEGG enrichment, and the results were visualized using a bioinformatics platform (Figure 3D,E). Through GO functional enrichment analysis, we identified 1682 terms with significant enrichment (*p* < 0.05). Most (1545) were biological processes (BP), with key functions including regulation of inflammatory responses, phosphorylation, and apoptotic signaling pathways. These directly correspond to EVO-induced renal inflammation, tubular epithelial cell injury, and cell death. For cellular components (CC), 111 terms were identified, mainly including mitochondrial membrane, ribonucleoprotein granule, and membrane rafts, suggesting that damage occurs at the subcellular level and is closely linked to disruption of tubular structure. Molecular function (MF) analysis yielded 26 terms, mainly including protein kinase binding, phosphatase binding, and peptidase activity, indicating that EVO mainly mediates downstream toxic signals by regulating kinase activity. The KEGG enrichment analysis identified 190 pathways, showing that EVO-induced nephrotoxicity was mainly associated with the TNF signaling pathway, inflammatory mediator regulation of TRP channels, the Apelin signaling pathway, and the PPAR signaling pathway. These pathways are involved in calcium overload, inflammation activation, lipid metabolism disorders, and endothelial dysfunction, respectively.

#### 2.3.3. Construction and Analysis of the “EVO-Target-Pathway” Network

The EVO–target–pathway network is shown in Figure 3F. The constructed interaction network comprises 142 nodes and 490 connecting edges, where the node size represents the Degree values. Key targets associated with EVO-induced renal injury, such as AKT1, TNF, TP53 and ALB, were enriched in prominent pathways that ranked highly in the KEGG analysis. These targets generated numerous hubs in inflammatory mediator regulation of TRP channels, the apelin signaling pathway, and the PPAR signaling pathway. This observation suggests a strong potential association between these pathways and the nephrotoxic effects induced by EVO.

### 2.4. Exploration of Differentially Expressed Genes (DEGs)

**Figure 4:**
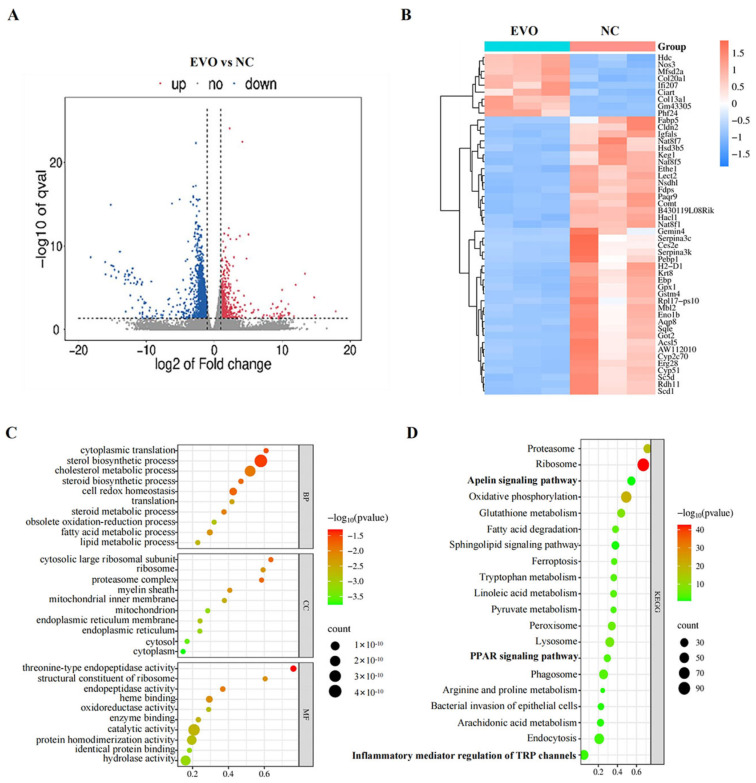
Differentially expressed gene analysis (DEGs) of the EVO-treated and control groups. (**A**) Volcano plots of the DEGs (red dots represent up-regulated genes, and blue dots down-regulated genes); (**B**) hierarchical clustering of DEGs (blue indicates low expression; white indicates moderate expression; red indicates high expression; NC-1/2/3 indicates three different kidney samples of the control group; EVO-1/2/3 indicates three different kidney samples of the EVO model group); (**C**) scatter plot of differentially expressed gene GO enrichment analysis (top 10); (**D**) scatter plot of differentially expressed gene KEGG pathway enrichment analysis (top 20).

Compared with the NC group, 2888 differentially expressed genes (DEGs) were detected in the EVOH group (*p* < 0.05), including 504 up-regulated genes and 2384 down-regulated genes (Figure 4A; Table S1). In this study, the number of down-regulated genes (2384) far exceeded that of up-regulated genes (504), a phenomenon consistent with the cytotoxic effects of EVO. EVO can lead to dysfunction and death of renal tubular epithelial cells by inhibiting the expression of genes related to cellular metabolism, proliferation, and survival. Meanwhile, the up-regulated genes were primarily concentrated in pathways associated with inflammatory responses and stress responses, representing the body’s compensatory reaction to damage. To identify distinct gene expression signatures between EVO-treated and NC groups, hierarchical clustering was performed based on log10 RPKM values (Figure 4B). Subsequently, GO functional annotation and KEGG pathway enrichment analyses were applied to the 2888 gene sets derived from the transcriptome. GO functional analysis yielded 1279 terms with statistical significance (*p* < 0.05), among which 725 were in the biological process (BP) category, mainly related to cellular redox balance, oxidative stress response, and cellular oxidative detoxification; 220 were in the cellular component (CC) category, mainly involving mitochondria, lysosomes, and ribosomes; and 334 were in the molecular function (MF) category, mainly including redox enzyme activity, hydrolase activity, and peroxidase activity (Figure 4C). GO functional analys. These results suggest that oxidative stress and mitochondrial dysfunction are core events in EVO-induced kidney injury. Enrichment analysis of the differentially expressed genes using the KEGG database revealed 312 enriched biological pathways, of which 74 were statistically significant (Table S2), including inflammatory mediator regulation of TRP channels, PPAR signaling pathway, apelin signaling pathway and other signaling pathways (Figure 4D). Notably, these pathways were also significantly enriched in a network toxicology framework using KEGG, highlighting their critical involvement in EVO-associated renal damage. This lays the foundation for subsequent integrated analysis of transcriptomics and network toxicology to identify core toxic targets and pathways.

### 2.5. Integration of Network Toxicology and Transcriptomics Analysis

**Figure 5:**
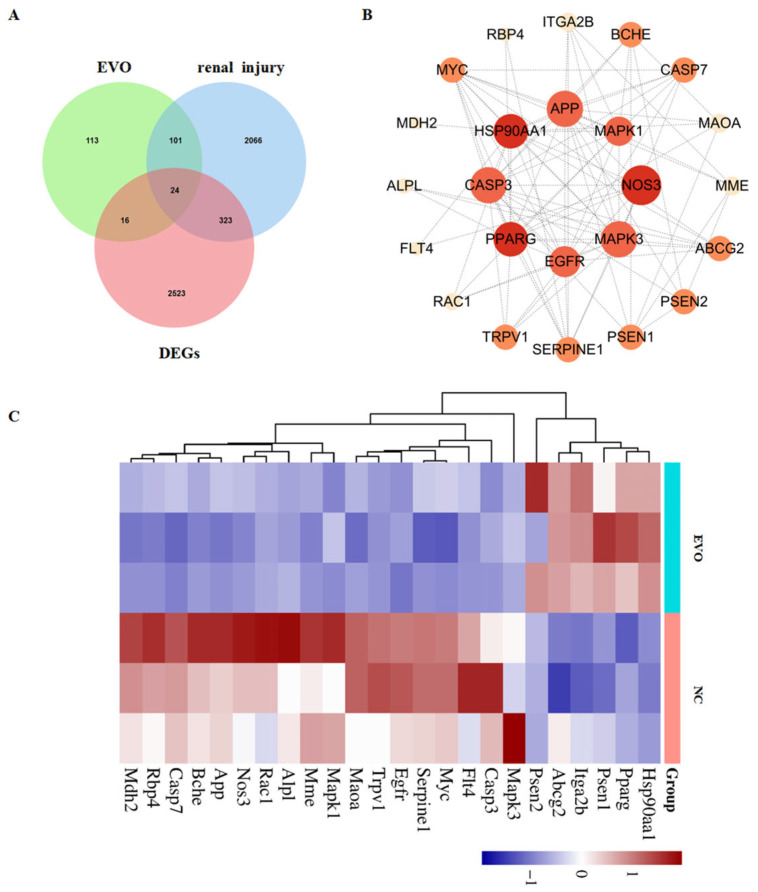
Network toxicology combined with transcriptomic analysis. (**A**) Target intersection Venn diagram of EVO, renal injury and DEGs; (**B**) PPI network diagram of EVO-induced nephrotoxic targets and DEGs; (**C**) hierarchical clustering of intersection genes (the color scale on the right represents gene expression levels, with red indicating high expression and blue indicating low expression).

**Figure 6:**
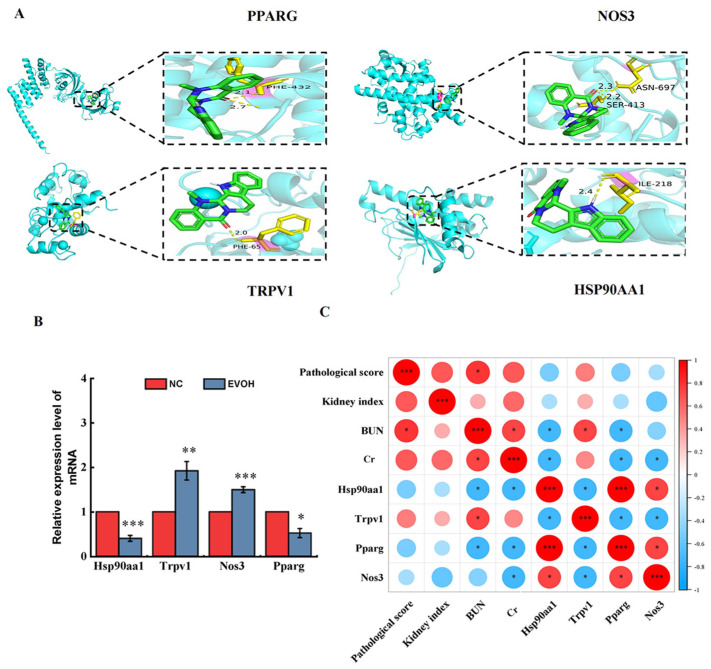
Validation of differentially expressed genes (DEGs). (**A**) Diagram of molecular docking model and active site; (**B**) the verification results of DEGs by qRT-PCR; (**C**) the Spearman correlation coefficient was calculated to analyze the relationships between renal function indicators, pathological score, kidney index and DEGs (red represents positive correlation, blue represents negative correlation). N = 6, * represents significance compared to the NC group, * indicated *p* < 0.05, ** indicated *p* < 0.01, *** indicated *p* < 0.001.

By integrating candidate targets from network toxicology with DEGs from transcriptome sequencing, 24 overlapping targets were identified (Figure 5A). To investigate protein–protein interactions among the 24 overlapping targets, we analyzed them using the STRING database and visualized the PPI network with Cytoscape 3.10.1 (Figure 5B). We also performed cluster analysis on the 24 overlapping targets; the heatmap is shown in Figure 5C. This study further selected four targets—TRPV1, NOS3, HSP90AA1, and PPARG—from the 24 overlapping targets for subsequent validation. The screening criteria included two main aspects: first, sorting by PPI network Degree values to select key targets with high node connectivity that occupy central positions in the regulatory network; second, combining literature evidence and functional data to select targets closely associated with kidney injury, inflammatory response, oxidative stress, and mitochondrial dysfunction. Four targets (TRPV1, NOS3, HSP90AA1, PPARG) were molecularly docked with EVO (Figure 6A). Molecular docking analysis showed that the binding energies of EVO to TRPV1, NOS3, HSP90AA1, and PPARG were −6.6, −8.04, −6.64, and −8.07 kcal/mol, respectively. A binding energy below −5.0 kcal/mol indicates high binding affinity and favorable binding activity [18]. Among these, PPARG showed the highest binding affinity (Table 1).

### 2.6. Validation of the Differentially Expressed Genes

We selected four DEGs associated with nephrotoxicity (*Pparg*, *Nos3*, *Hsp90aa1* and *Trpv1*) for validation by qRT-PCR (Figure 6B). The results verified by qRT-PCR confirmed that, compared with the blank control group, the mRNA expression levels of *Trpv1* and *Nos3* were significantly up-regulated by approximately 1.92 times and 1.50 times respectively (*p* < 0.01, *p* < 0.001), while the expression levels of *Hsp90aa1* and *Pparg* were significantly down-regulated by approximately 59.7% and 47.7% respectively (*p* < 0.05, *p* < 0.001). The expression trends were consistent with transcriptome sequencing results, fully validating the reliability, stability, and reproducibility of the transcriptome data. Further Spearman correlation analysis showed that *Trpv1* expression was significantly positively correlated with renal function markers (BUN, Cr), renal index, and pathological injury score, indicating that high *Trpv1* expression is closely associated with the severity of renal injury; expression levels of *Nos3*, *Pparg*, and *Hsp90aa1* showed significant negative correlations, suggesting that down-regulation of these three genes directly contributes to EVO-induced renal injury and tissue damage. The correlations between molecular expression and renal function/pathological injury further confirm that these genes are key regulators of EVO-induced nephrotoxicity.

### 2.7. Investigating the Effect of EVO on Renal-Associated Protein Expression in Mice

**Figure 7:**
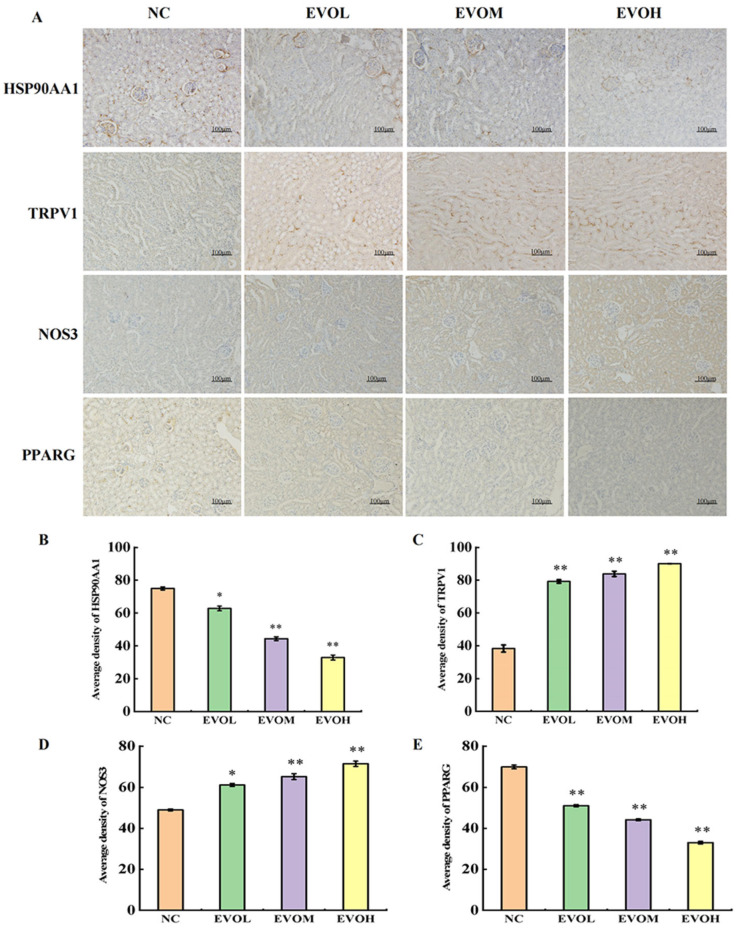
Effects of EVO on the expression levels of key proteins. (**A**) Immunohistochemical representative images of HSP90AA1, TRPV1, NOS3 and PPARG. (**B**–**E**) Statistical results of protein expression levels of HSP90AA1, TRPV1, NOS3 and PPARG. Note: NC: normal control group; EVOL: EVO low-dose group (10 mg/kg); EVOM: EVO medi-um-dose group (20 mg/kg); EVOH: EVO high-dose group (40 mg/kg). N = 6, * represents significance compared to the NC group, * indicated *p* < 0.05, ** indicated *p* < 0.01.

Protein expression levels of HSP90AA1, TRPV1, NOS3, and PPARG were evaluated by immunohistochemistry (IHC). The results are shown in Figure 7A–E. In comparison to the NC control group, the positive expression areas of TRPV1 and NOS3 showed a dose-dependent significant increase: in the EVOH, group the positive expression area of TRPV1 was 2.35 times that of the control group, and the positive expression area of NOS3 was 1.46 times that of the control group (*p* < 0.05). The positive expression areas of HSP90AA1 and PPARG showed a dose-dependent significant decrease. In the EVOH group, the positive expression areas of HSP90AA1 and PPARG were 0.44 times and 0.47 times higher than those in the control group, respectively (*p* < 0.05). Protein expression trends of the four key genes were completely consistent with mRNA levels, with no significant differences in post-transcriptional regulation, indicating that EVO mainly regulates expression of these targets at the transcriptional level. HSP90AA1 and NOS3 are mainly localized in the glomerulus, while TRPV1 and PPARG are mainly localized in the renal tubules, showing clear tissue distribution specificity. HSP90AA1 and NOS3 located in the glomerulus are mainly involved in maintaining glomerular structure, endothelial function, and oxidative stress regulation. Abnormal expression of HSP90AA1 can directly lead to impaired glomerular filtration function; TRPV1 and PPARG located in the renal tubules mainly regulate apoptosis, inflammatory response, and lipid metabolism of renal tubular epithelial cells. Disruption of their expression can exacerbate renal tubular injury, necrosis, and inflammatory infiltration. This glomerulotubular zonal regulation pattern further elucidates the molecular mechanism of EVO-induced kidney injury via multi-target, multi-regional synergistic effects at the tissue-specific level.

## 3. Discussion

Evodiamine (EVO) is an alkaloid with extensive pharmacological activities, so it has potential application value in anti-obesity, anti-diabetes, anti-inflammatory, and anti-tumor fields [6]. However, different doses of EVO significantly affect its efficacy and safety. In a colorectal cancer mouse model, 10 mg/kg EVO exerted anti-tumor effects by reshaping gut microbiota and alleviating intestinal inflammation [19]. In the Lung Cancer Metastasis model, 10 and 20 mg/kg EVO reduced tumor nodules, but high-dose EVO caused significant body weight loss [20]. In a post-traumatic stress disorder model, 10 mg/kg EVO exerted significant neuroprotective effects on hippocampal neurons, whereas the efficacy of 20 mg/kg EVO was reduced [21]. Further studies found that adding 1 mg/kg EVO to the diet could prevent obesity and insulin resistance in mice. However, 10 mg/kg EVO caused complications such as cardiac hypertrophy [22]. Despite its wide-ranging therapeutic promise, the clinical translation of EVO is hindered by its associated toxicities. Many studies have confirmed that EVO can induce cardiotoxicity, hepatotoxicity, and nephrotoxicity [10,11,23]. Among these, studies on nephrotoxicity are relatively limited. One study reported that intragastric administration of 5 g/kg EVO in mice activates TRPV1 protein and induces calcium overload. Subsequently, calcium overload induced apoptosis through the PI3K pathway, eventually leading to nephrotoxicity [10]. However, this dosage is significantly higher than clinical dosages. To more accurately assess the nephrotoxic risk of EVO, further investigation is needed. This study established three dose groups: 10, 20, and 40 mg/kg. This design covers the clinically relevant dose range and clarifies the toxicity threshold through dose–response analysis. Therefore, in this study, we evaluated nephrotoxicity risk by administering EVO orally to mice at different doses, integrated network toxicology and transcriptomics to explore the underlying toxic mechanism, and validated key targets using molecular docking.

Creatinine (Cr) and uric acid (UA) are widely recognized as clinical biomarkers for evaluating renal function. Elevated levels indicate the degree of renal cell injury [24]. Previous studies have shown that intragastric administration of 5 g/kg EVO for 14 days causes degenerative lesions in renal tubules, thickening of the renal tubular basement membrane, stromal monocyte infiltration, and significantly elevated UA levels [10]. Similarly, in our experiment, the 40 mg/kg dosage group exhibited disorganization of glomerular structure after 14 days of treatment, along with dilation of the renal tubule lumens accompanied by inflammatory cell infiltration. After 28 days, the lesions were further aggravated, showing renal tubular swelling, cell edema and necrosis, accompanied by significantly increased Cr and BUN levels. The kidneys are important metabolic and excretory organs in the body. Piao et al. [25] reported that changes in renal index are often more sensitive toxicity endpoints than body weight changes. The present results demonstrated that renal index in EVO-treated mice was significantly increased in a dose-dependent manner; Meanwhile, body weight decreased in a dose-dependent manner. Previous studies have confirmed through database retrieval and regression analysis that there is no direct correlation between changes in absolute kidney weight and organ index and changes in body weight [10]. Based on the present findings, it is further confirmed that EVO-induced kidney injury is a direct and specific effect of EVO, rather than an indirect effect caused by non-specific factors such as weight loss and systemic malnutrition. Although the renal damage observed in the 10 mg/kg and 20 mg/kg dosage groups was relatively mild, they still exhibited nephrotoxicity with prolonged exposure time and increased dose. Additionally, the study found that EVO significantly inhibited weight gain in mice, and this effect was particularly significant after 20 days of administration, which was consistent with previous reports [10]. These findings indicate that EVO-induced nephrotoxicity is dose- and time-dependent. Even at a relatively low dose, long-term exposure may still carry the risk of nephrotoxicity.

Network toxicology aims to construct computational network models to comprehensively characterize toxic effects and their underlying mechanisms [26]. Transcriptomics enables the comparison of gene expression profiles between cells or tissues under distinct physiological conditions or disease states, thereby identifying alterations in gene activity [27]. Qu et al. [28] integrated network toxicology with transcriptomic profiling to identify marked changes in gene expression and critical cellular pathways associated with 2,2′,4,4′-tetrabromodiphenyl ether-induced neuronal damage. This study identified 125 overlapping targets between EVO-related targets and kidney injury-related targets. Previous studies have shown that there are 45 overlapping targets for aristolochic acid I-induced kidney injury and 62 for *Tripterygium wilfordii*-induced kidney injury [29,30]. The number of overlapping targets obtained here is consistent with those in similar natural product toxicity studies, further confirming that EVO nephrotoxicity results from the combined action of multiple targets. Such a large number of targets also suggests that single-target intervention may be difficult to completely block the nephrotoxicity of EVO. After in-depth analysis, four core targets closely related to nephrotoxicity (PPARG, NOS3, HSP90AA1, TRPV1) were selected for molecular docking. Molecular docking results showed that EVO has strong binding affinity for these four proteins, suggesting that they play key roles in EVO-induced nephrotoxicity. KEGG enrichment analysis revealed that inflammatory mediator regulation of TRP channels, the PPAR signaling pathway, the apelin signaling pathway, and other pathways play pivotal roles in EVO-induced nephrotoxicity.

Heat shock proteins (HSPs) are important stress proteins often used as biomarkers for various types of stress [31]. These molecules are classified into distinct families, including HSP90, HSP70, HSP60, HSP40, and small heat shock proteins (sHSPs) [32]. Among these, HSP90 is primarily expressed within the podocytes and mesangial cells that constitute the glomerulus [33]. In functionally deficient tissues, HSP90 can mediate autophagy, necrotic apoptosis, and ferroptosis [34]. Studies have demonstrated that HSP90AA1 exerts anti-inflammatory effects by decreasing pro-inflammatory cytokine levels and inhibiting cell apoptosis [35]. Zhang et al. [36] reported that in cisplatin-induced acute kidney injury, HSP90AA1 was significantly down-regulated. After down-regulating *Hsp90aa1*, the expression levels of pro-apoptotic proteins Caspase-3 and Bax were up-regulated, whereas the anti-apoptotic protein Bcl-2 was down-regulated, leading to an overall increase in cellular apoptosis. Similarly, in the present study, mRNA and protein levels of *Hsp90aa1* in mouse kidney tissue were significantly down-regulated after EVO treatment. Combined with the results of HE staining, local necrosis was found in renal tubules. Therefore, we speculate that reduced *Hsp90aa1* expression weakens the anti-inflammatory and anti-apoptotic capacity of kidney cells, thereby enhancing inflammatory responses and apoptosis and ultimately contributing to EVO-induced kidney injury.

As essential nuclear transcription factors, PPARs exert significant influence over lipid metabolism and inflammation; this family of receptors comprises three distinct subtypes: PPAR-α, PPAR-β, and PPAR-γ [37,38]. Among them, PPARγ (PPARG) regulates lipid metabolism and inhibits renal inflammation, acting as a key molecule in anti-inflammatory and immune regulation [39,40]. Previous studies have confirmed that PPARG agonists (GW1929) attenuate inflammasome formation and apoptosis induced by mercuric chloride by regulating Bcl2 and NF-κB signaling in human renal tubular epithelial cells, whereas PPARG inhibition significantly exacerbates mercuric chloride-induced nephrotoxicity in these cells [41]. Another study showed that mangiferin can activate PPARG, thereby inhibiting the expression of inflammatory factors such as NF-κB and exerting a protective effect on the kidneys. PPARG*g* has been shown to protect against renal injury, including glomerulosclerosis, glomerulonephritis, and interstitial inflammation, by regulating inflammatory pathways [42]. Gan et al. [43] reported that silibinin reduces ROS accumulation induced by avermectin via PPARG and modulates inflammation, oxidative stress, and ferroptotic damage in carp kidney tissue. In this study, 25 DEGs were significantly enriched in the PPAR signaling pathway, including 4 significantly up-regulated genes and 21 significantly down-regulated genes; *Pparg* showed the most significant down-regulation (Table S3). Meanwhile, molecular docking results confirmed that EVO has the highest binding affinity for *Pparg*, indicating that *Pparg* is a key target of EVO. In addition, this study observed significantly reduced mRNA and protein levels of *Hsp90aa1* in mouse kidney tissue after EVO treatment. Consistent with HE staining, focal tubular necrosis was observed. We further speculate that inhibition of the PPARγ signaling pathway and reduced *Hsp90aa1* expression both contribute to EVO-induced renal tubular injury. Therefore, we further hypothesize that *Pparg* and *Hsp90aa1* exert a synergistic effect in the process of EVO-induced kidney injury: on the one hand, EVO inhibits PPARG, weakening renal anti-inflammatory and metabolic regulatory capacity and triggering inflammatory signals; on the other hand, it down-regulates HSP90AA1, impairing the stress-protective function of kidney cells and rendering them unable to counteract inflammation and apoptotic damage effectively. Together, these two events promote aggravated renal inflammatory infiltration and exacerbated tissue damage, thereby jointly mediating EVO-induced nephrotoxicity.

Transient receptor potential (TRP) channels constitute a group of non-selective cation channels, which play an important role in many physiological activities and pathological processes [44]. Studies have shown that the activation of TRP channels can create conditions favorable for the activation of inflammatory cells [45]. Zhong et al. [46] reported that TRPV1 knockout enhanced renal macrophage infiltration and collagen deposition in mice fed a Western diet. Within the TRP channel family, *Trpv1* plays a crucial role in thermoregulation, circadian rhythms, acute and chronic inflammation, and mediates calcium overload and apoptosis [47]. Yang et al. [10] reported that EVO induces intracellular calcium overload and apoptosis by activating TRPV1. Treatment with a TRPV1 antagonist and calcium chelator significantly attenuated EVO-induced weight loss and renal damage. In this study, we also observed significantly up-regulated *Trpv1* expression, consistent with previous findings [10]; its expression was significantly positively correlated with renal function markers and pathological injury severity, further confirming that *Trpv1* is a key target mediating EVO-induced nephrotoxicity.

Apelin, as a potent angiogenic factor, stimulates the release of nitric oxide (NO), leading to vasodilation and enhanced myocardial contractility [48,49,50]. Studies have shown that apelin attenuates renal ischemia/reperfusion injury in diabetic rats and decreases inflammatory factor levels in serum and kidney tissue [51]. Endothelial nitric oxide synthase, also termed eNOS (NOS3), is a key isoenzyme: it not only lowers vascular tone and regulates endothelium-dependent vasodilation, but also safeguards glomeruli against sclerosis and structural impairment [52]. In this study, we found that in addition to *Nos3*, 24 other related DEGs were significantly enriched in the apelin signaling pathway. Among these, 7 genes exhibited significant up-regulation, while 17 genes were significantly down-regulated (Table S4). Accumulating evidence suggests that the regulation of nitric oxide (NO) production is a critical mechanism for preserving endothelial cell homeostasis and function [53]. Combined with the significant up-regulation of *Nos3* observed in this study, we speculate that EVO may modulate NO release via the apelin signaling pathway, thereby damaging renal endothelial cells and ultimately inducing nephrotoxicity. This mechanism is likely one of the important factors contributing to the pathological changes in renal tissues.

This study has several limitations. The primary limitation is the lack of direct functional validation for the identified targets and pathways. Our findings are largely based on correlational data from transcriptomics and expression verification. To establish a causal link between targets such as *Pparg*, *Hsp90aa1*, *Nos3*, and *Trpv1* and EVO-induced nephrotoxicity, further in vivo and in vitro experiments are required. For example, specific agonists or antagonists (e.g., a PPARG agonist or TRPV1 antagonist) could be used to determine whether they rescue the renal injury phenotype. Alternatively, siRNA-mediated knockdown of these key genes in renal cell lines (e.g., HK-2) could be performed to confirm their functional roles. Secondly, transcriptomic profiling was exclusively conducted on samples obtained from the high-dose group at a single time point (day 28). This may not capture the full spectrum of molecular changes, as the toxic mechanism may differ at lower doses or earlier stages. The lack of a dynamic molecular analysis alongside the time-course pathological examination is another constraint. The specific functions and interactions of these genes remain poorly understood, and their upstream and downstream regulatory networks have not been fully elucidated. Thus, future studies are needed to further elucidate the molecular mechanisms whereby these pathways and genes mediate EVO-induced nephrotoxicity and to develop targeted agonists/antagonists as potential strategies to alleviate EVO-induced nephrotoxicity.

## 4. Materials and Methods

### 4.1. Drug and Animal Treatment

Evodiamine (EVO) powder (purity > 98%) was purchased from Tianjin Kemiou Chemical Reagent Co., Ltd. (Tianjin, China). In total, 100 mg EVO was dissolved and suspended in 0.5% carboxymethyl cellulose sodium (CMC-Na) solution. Three EVO suspensions were prepared at concentrations of 10 mg/kg, 20 mg/kg, and 40 mg/kg. Suspensions were prepared using a 100 MHz ultrasonic device (Kunshan Ultrasonic Instruments Co., Ltd., Kunshan, China) to ensure full dispersion. It was stored in a refrigerator at 4 °C for future use. The EVO suspension needs to be re-prepared once a week and can be stored at 4 °C for 7 days.

Male KM mice (5–6 weeks, weighing 20 ± 2 g) were supplied by Guizhou Huiqiu Biological Technology Co., Ltd. (Guizhou, China; License No. SCXK(Yu)2020-0005). All animal procedures were approved by the Animal Experiment Ethics Committee of Guizhou University of Traditional Chinese Medicine (Ethics Approval No. 20240192) and performed in accordance with the NIH Guide for the Care and Use of Laboratory Animals.

After 1 week of acclimatization, mice were randomly assigned to four groups (n = 20 per group): normal control (NC), EVO low-dose (EVOL), EVO medium-dose (EVOM), and EVO high-dose (EVOH) groups. The EVO groups received intragastric administration of EVO at 10, 20, and 40 mg/kg, with a gavage volume of 0.3 mL/10 g. The NC group received an equal volume of CMC-Na solution. This model was established to investigate EVO-associated nephrotoxicity. All animals underwent daily intragastric dosing for the duration of the experiment. Body weight was measured every 4 days to monitor growth changes. Treatment was maintained for 28 days, and samples were collected on days 7, 14, and 28; all mice were anesthetized with a 0.5% (*w*/*v*) pentobarbital sodium solution. Orbital blood samples were harvested and subsequently centrifuged. Mice were euthanized by cervical dislocation. Kidney tissues were immediately collected, weighed, and used to calculate the renal index.

### 4.2. Serum Biochemical Indexes

Blood samples were collected from the mice in each group and transferred into centrifuge tubes. After centrifugation at 3500 *g* for 10 min at 4 °C, the supernatant (serum) was carefully collected. Serum levels of blood urea nitrogen (BUN) and creatinine (Cr) were measured according to the manufacturer’s instructions. BUN kit (Nanjing Jiancheng Bioengineering Institute, Cat. No.: C013-2-1); Cr kit (Nanjing Jiancheng Bioengineering Institute, Cat. No.: C011-2-1).

### 4.3. Renal Pathological Examination

An appropriate amount of renal tissue was collected, washed with physiological saline to remove surface blood contamination, and then fixed in 4% paraformaldehyde solution. The tissue sections were then subjected to Hematoxylin and Eosin (HE) staining for histological analysis, which included the main steps of dehydration, embedding, sectioning, staining and resin sealing. Finally, these parts were examined using an optical microscope. According to references [54,55], the severity and scope of pathological damage were graded and scored based on five indicators: glomerular basement membrane thickening, renal tubular dilation, hypertrophy of tubular epithelial cells, tubular epithelial degeneration and necrosis, and inflammatory cell infiltration. The grading scale was divided into four levels: no injury (<5%, 0 points); mild injury (5–25%, 1 point); moderate injury (26–50%, 2 points); and severe injury (>50%, 3 points). A cumulative histopathological injury score was determined for each renal tissue specimen and analyzed statistically.

### 4.4. Network Toxicology Study of EVO-Induced Renal Injury in Mice

#### 4.4.1. Prediction of Targets for EVO and Renal Injury

The structural formula of EVO was obtained from the PubChem database (https://pubchem.ncbi.nlm.nih.gov/, accessed on 10 August 2024). Its potential molecular targets were predicted by integrating multiple public databases: the Traditional Chinese Medicine Systems Pharmacology Database (TCMSP, https://tcmsp-e.com/tcmsp.php, accessed on 10 August 2024), SwissTargetPrediction (https://www.swisstargetprediction.ch/, accessed on 10 August 2024), TargetNet (http://targetnet.scbdd.com/home/index/, accessed on 10 August 2024), Comparative Toxicogenomics Database (CTD, https://ctdbase.org/, accessed on 10 August 2024), and Similarity Ensemble Approach (SEA) database (https://sea.bkslab.org, accessed on 10 August 2024) [16]. The target lists from these five databases were merged and deduplicated. Using the keyword “renal injury”, we retrieved relevant targets from the GeneCards database (https://genecards.org/, accessed on 10 August 2024).

#### 4.4.2. Constructing the Protein–Protein Interaction (PPI) Network and Identifying Core Targets

Overlapping targets between EVO and renal injury were identified using the Venny 2.1.0 tool (http://liuxiaoyuyuan.cn/, accessed on 3 September 2024). The intersecting targets were subsequently imported into the STRING platform (https://string-db.org/, accessed on 3 September 2024) to retrieve information on protein–protein interactions. The PPI file from STRING was imported into Cytoscape 3.10.1 (https://cytoscape.org/, accessed on 3 September 2024) to construct the network, with node size and color encoding the Degree value to facilitate the identification of key targets.

#### 4.4.3. Gene Ontology (GO) Functional Enrichment Analysis and Kyoto Encyclopedia of Genes and Genomes (KEGG) Pathway Enrichment Analysis

The intersecting targets were imported into Metascape (http://metascape.org, accessed on 6 October 2024) to conduct both GO functional enrichment and KEGG pathway enrichment analyses. Pathways were prioritized by *p*-value, and the top 10 biological processes (BP), cellular components (CC), molecular functions (MF), and top 20 signaling pathways were selected. Visualization was performed using the Weishengxin tool (https://www.bioinformatics.com.cn/, accessed on 6 October 2024).

#### 4.4.4. Construction of the “EVO-Target-Pathway” Network

Key targets related to EVO-induced renal injury, together with the top 20 pathways and their related targets, were uploaded to Cytoscape 3.10.1 to construct the EVO–target–pathway network.

#### 4.4.5. Molecular Docking

The three-dimensional structure of EVO was obtained from the PubChem database, and the PDB files of key targets were retrieved from the RCSB PDB database (https://www.rcsb.org/, accessed on 13 November 2024). Target proteins were processed for hydrogenation and water removal using AutoDockTools 1.5.6 (https://autodock.scripps.edu, accessed on 13 November 2024). The receptor and ligand PDBQT files were imported into AutoDockTools 1.5.6 to define the molecular docking region. After molecular docking, visualization was performed using PyMOL (Version 2.4.0) software (open-source, https://pymol.org, accessed on 25 November 2024).

### 4.5. RNA Extraction and Transcriptome Sequencing

RNA extraction and sequencing were performed by Lianchuan Biotechnology Co., Ltd. (Hangzhou, China). Samples from the EVOH group and the NC group at day 28 were selected for transcriptome sequencing. This experimental procedure was performed as described in our previously published study [26]. Total tissue RNA was extracted using the TRIzol method. RNA purity was determined using a NanoPhotometer^®^ spectrophotometer (Implen GmbH, Munich, Germany), and RNA integrity was verified by agarose gel electrophoresis. mRNA was enriched using Oligo (dT) magnetic beads, fragmented, and reverse-transcribed into double-stranded cDNA. Sequencing adapters were ligated, followed by PCR amplification to construct a strand-specific cDNA library. Qualified libraries were sequenced on the Illumina NovaSeq 600 platform (San Diego, CA, USA) to generate paired-end 150 bp reads, with a sequencing depth of no less than 6 Gb clean data per sample. Raw data were filtered using Cutadapt software (version 3.4) to remove low-quality sequences, adapter sequences, and redundant sequences. Clean reads were mapped to the reference genome using HISAT2 software (version 2.2.1). Gene expression levels were quantified by RPKM values and normalized using the DESeq2 method. Differentially expressed genes (DEGs) were identified under the thresholds of |log2FC| ≥ 1 and *p* < 0.05, followed by GO functional annotation and KEGG pathway enrichment analysis.

### 4.6. Real-Time Quantitative Polymerase Chain Reaction (qRT-PCR) Analysis

Four genes (*Pparg*, *Nos3*, *Hsp90aa1*, and *Trpv1*) were selected for qRT-PCR validation. Primers were designed and synthesized by Wuhan Huarun Biotechnology Co., Ltd. (Wuhan, China) (Table 2). The qRT-PCR experimental protocol was performed in accordance with the method described in our previous study [25]. Total RNA was extracted from kidney tissues using TRIzol, and cDNA was synthesized using a reverse transcription kit. qRT-PCR was conducted on a real-time PCR system. The reaction process was as follows: 10 min of pre-denaturation at 95 °C (1 cycle), 15 s of denaturation at 95 °C, and 60 s of annealing and extension at 60 °C (40 cycles). All experiments involved three biological replicates and three technical replicates. Relative gene expression was calculated using the 2^−ΔΔCt^ method and normalized to the reference gene. Each value represents the average ± standard deviation (SD), and the results were statistically analyzed using SPSS 27.0 software (SPSS Inc., Chicago, IL, USA).

### 4.7. Immunohistochemistry

Immunohistochemistry was performed to assess HSP90AA1, TRPV1, NOS3 and PPARG expression in kidney sections. After dewaxing and antigen retrieval, sections were blocked with 5% bovine serum albumin (AR0004, Boster, Wuhan, China) and incubated at 37 °C for 30 min. The primary antibodies HSP90AA1 (1:200, HUABIO, Woburn, MA, USA, ET1605-57); TRPV1 (1:500, Biological Industries, Sydney, NSW, Australia, bs-23926R); NOS3 (1:400, Boster, Wuhan, China, A01604-2); PPARG (1:500, Bioss, Woburn, MA, USA, bsm-33436M) were incubated overnight at 4 °C. The next day, sections were washed with PBS, incubated with HRP-conjugated secondary antibody (1:10,000; Boster, Wuhan, China, BA1054) at 37 °C for 30 min. Immunohistochemical staining was carried out according to what was described in our previous research [56]. Positive signals were visualized by DAB staining (reagent model AR1027, purchased from Boster Company in Wuhan, China), followed by counterstaining with hematoxylin; positive signals appeared as brown precipitates under a light microscope. In the experiment, three non-overlapping 200× magnified fields of view were randomly selected. Image-Pro Plus 6.0 software (version 1.54i, National Institutes of Health, USA) was used to analyze the average staining intensity and calculate the positive expression rate based on the mean grayscale value. During the operation, three independent fields of view were repeatedly selected, and the expression rate was quantitatively calculated using ImageJ.

### 4.8. Statistical Analysis

Data processing was conducted using Excel 2019 (Microsoft, Redmond, WA, USA) and Origin 2021 software (OriginLab Inc., Northampton, MA, USA). Statistical analysis was conducted using SPSS 27.0 software (SPSS Inc.). Student’s *t*-test was employed to make statistical comparisons between the two groups. For comparisons involving more than two groups, one-way analysis of variance (ANOVA) was used, followed by Tukey’s post hoc test. The data are presented as mean ± standard deviation (SD), and *p* < 0.05 was considered statistically significant.

## 5. Conclusions

This study innovatively combines network toxicology and transcriptomics to systematically reveal the toxic regulatory network of evodiamine at the multi-target, multi-pathway level. The results indicate that *Pparg*, *Nos3*, *Hsp90aa1*, and *Trpv1* are the four core genes mediating renal injury. Evodiamine inhibits the expression of *Pparg* and *Hsp90aa1*, reduces renal anti-inflammatory and metabolic regulatory capacity, weakens the stress-protective function of renal cells, and activates inflammatory signals; It simultaneously activates *Trpv1* to trigger calcium overload, interferes with the apelin signaling pathway, causes abnormal NO production and renal vascular damage, and ultimately induces nephrotoxicity through the synergistic effects of multiple pathways. This study provides preliminary insights into the potential mechanism of EVO-induced nephrotoxicity and lays a foundation for further research on the safe clinical application of evodiamine.

## Figures and Tables

**Table 1:** The binding energies of EVO and DEGs.

Target	Target (PDB ID)	Affinity (kcal/mol)
HSP90AA1	3b27	−6.64
NOS3	3N5W	−8.04
PPARG	6FZY	−8.07
TRPV1	3SUI	−6.6

**Table 2:** Sequences of the quantitative real-time PCR primers.

Gene	Primer	Sequence (5′-3′)
*Gapdh*	Forward Primer	ATGGCCTTCCGTGTTCCTAC
	Reverse Primer	AAGTCGCAGGAGACAACCTG
*Hsp90aa1*	Forward Primer	GGCAGAGGCTGACAAGAATG
	Reverse Primer	TCCTGTTAGCATGGGTCTGG
*Trpv1*	Forward Primer	TGCTTCAGGGTGGATGAGGT
	Reverse Primer	CTCCCTGAAACTCGGCCTG
*Nos3*	Forward Primer	TCACTATGGCAACCAGCGTC
	Reverse Primer	AAGAAAAGCTCTGGGTGCGT
*Pparg*	Forward Primer	GACCACTCGCATTCCTTT
	Reverse Primer	CCACAGACTCGGCACTCA

## Data Availability

The original contributions presented in this study are included in the article/Supplementary Material. Further inquiries can be directed to the corresponding author.

## Supplementary Materials

The following supporting information can be downloaded at: https://www.mdpi.com/article/10.3390/ijms27093793/s1.

## Abbreviations

EVO	Evodiamine
CMC-Na	Carboxymethyl cellulose
BUN	blood urea nitrogen
Cr	creatinine
HE	Hematoxylin and Eosin
PPI	Protein–Protein Interactions
GO	*Gene Ontology*
KEGG	Kyoto Encyclopedia of Genes and Genomes
BP	Biological Processes
CC	Cellular Components
MF	Molecular Function
DEGs	Differentially Expressed Genes
